# AChRAb and MuSKAb double-seropositive myasthenia gravis: a distinct subtype?

**DOI:** 10.1007/s10072-021-05042-3

**Published:** 2021-01-12

**Authors:** Jieni Zhang, Yin Chen, Jiaxin Chen, Xin Huang, Haiyan Wang, Yan Li, Weibin Liu, Huiyu Feng

**Affiliations:** Department of Neurology, The First Affiliated Hospital, Sun Yat-sen University, Guangdong Provincial Key Laboratory of Diagnosis and Treatment of Major Neurological Diseases, No.58 Zhongshan 2nd Road, Guangzhou, 510080 China

**Keywords:** Myasthenia gravis, Antibody, Acetylcholine receptor, Muscle-specific tyrosine kinase, Double-seropositive myasthenia gravis, Subtype

## Abstract

**Introduction:**

This study investigated the characteristics of double-seropositive myasthenia gravis (DSP-MG) in southern China for disease subtype classification.

**Methods:**

A case-control study was carried out in which the characteristics of DSP-MG patients (*n* = 17) were compared to those of muscle-specific tyrosine kinase antibody-positive (MuSK)-MG and acetylcholine receptor antibody-positive (AChR)-MG patients (*n* = 8 and 27, respectively). We also performed a literature review of DSP-MG patients.

**Results:**

Compared to AChR-MG, DSP-MG had greater bulbar dysfunction (47.1% vs 18.6%, *P* = 0.04), higher incidence of myasthenia crisis (41.2% vs 14.8%, *P* = 0.04), more severe Myasthenia Gravis Foundation of America classification at maximum worsening, greater autoantibody abnormalities (70.6% vs 33.3%, *P* = 0.015), greater need for immunosuppressant treatment (58.8% vs 3.7%, *P* < 0.001), and worse prognosis with less remission (11.8% vs 55.6%, *P* = 0.001). There were no differences between DSP-MG and MuSK-MG patients. DSP-MG described in published reports was comparable to MuSK-MG.

**Discussion:**

DSP-MG in southern China may be a subtype of MuSK-MG.

## Introduction

Myasthenia gravis (MG) is an autoimmune disease involving 3 autoantibodies—namely, acetylcholine receptor antibody (AChRAb), muscle-specific tyrosine kinase antibody (MuSKAb), and low-density lipoprotein receptor-associated protein 4 antibody [1]. These are useful for identifying different subsets of MG patients as a prerequisite for accurate diagnosis and effective disease management. Previous studies have reported that in a small proportion of cases, AChRAb and MuSKAb coexist [1, 2]; however, it is unclear whether double-seropositive (DSP)-MG is more similar to AChR-MG or MuSK-MG or is a distinct clinical entity. In order to correctly classify and provide treatment tailored to DSP-MG patients, we compared the characteristics of AChR-MG, MuSK-MG, and DSP-MG cases at our hospital.

## Methods

### Subjects

This retrospective case-control study was conducted at the First Affiliated Hospital of Sun Yat-sen University between 2015 and 2018. Inclusion criteria were as follows: (1) patients diagnosed with MG based on typical clinical presentation and/or examination; (2) positivity for AChRAb, MuSKAb, or both; and (3) follow-up at least 2 years. Exclusion criteria were as follows: (1) patients with severe heart, lung, or kidney disease or multivisceral failure; (2) incomplete data; and (3) age < 18 years old. The study was approved by the Ethics Committee of the First Affiliated Hospital of Sun Yat-sen University.

### Methods

Patients were grouped according to antibody specificity as follows. AChRAb-positive patients were assigned to the AChR-MG group; MuSKAb-positive patients were assigned to the MuSK-MG group; and patients positive for both AChRAb and MuSKAb constituted the DSP-MG group. The following information was collected for each patient: sex; age of MG onset; clinical stage at onset; Myasthenia Gravis Foundation of America (MGFA) classification at maximum worsening; bulbar dysfunction; myasthenia crisis; thymus imaging; electrophysiology; thyroid function; autoantibodies, including anti-double-stranded DNA, anticentromere, anti-extractable nuclear antigen (Smith protein, ribonucleoprotein, Robert antigen/Sjögren’s A, and Lane antigen/Sjögren’s B), antiribosome, antinucleosome, antihistone, antimitochondrion, and antinuclear antibodies; response to treatment; and prognosis (based on prognostic immune score [PIS]).

### Autoantibody assay

AChRAb, MuSKAb, and other antibodies were detected by enzyme-linked immunosorbent assay (ELISA) with commercial ELISA kits (RSR, Cardiff, UK). The experiment was performed at Guangzhou Da’an Clinical Test Center. Patients were defined as antibody positive for antibody titers of ≥ 0.45 nmol/l (AChRAb) and > 9.5 pmol/l (MuSKAb).

### Statistical analysis

Data analysis was performed using SPSS v23.0 (SPSS Inc., Chicago, IL, USA). Quantitative data were assessed with the independent samples *t* test, and qualitative data were evaluated with the chi-square test, Fisher’s exact test, or rank sum test. For all tests, *P* ≤ 0.05 was considered statistically significant.

## Results

### Characteristics of DSP-MG vs AChR-MG

We compared the characteristics of 17 DSP-MG, 8 MuSK-MG, and 27 AChR-MG patients (Table 1). DSP-MG patients had greater bulbar palsy dysfunction, a higher incidence of myasthenia crisis, higher MGFA classification at maximum worsening, more autoantibody abnormalities, greater need for immunosuppressants, and a lower rate of pharmacologic remission. DSP-MG was more prevalent in females and was less frequently of the ocular type compared to AChR-MG, although the differences were nonsignificant.

**Table 1:** Characteristics of AChR-MG, MuSK-MG, and DSP-MG patients

	AChR-MG (*n* = 27)	MuSK-MG (*n* = 8)	DSP-MG (*n* = 17)	P1 value (AChR-MG vs DSP-MG)	P2 value (MuSK-MG vs DSP-MG)
Age, years	37.24 ± 14.07	39.24 ± 14.65	40.76 ± 14.33	0.539	0.734
Age of onset, years	35.37 ± 18.25	38.63 ± 16.91	38.41 ± 18.00	0.591	0.978
0–18, *n* (%)	5 (18.5)	1 (12.5)	2 (11.8)	0.910	0.825
19–50, *n* (%)	16 (59.3)	4 (50.0)	11 (64.7)		
>50, *n* (%)	6 (22.2)	3 (37.5)	4 (23.5)		
Sex, *n* (%)				0.109	1.000
Male	11 (40.7)	1 (12.5)	3 (17.6)		
Female	16 (59.3)	7 (87.5)	14 (82.4)		
Clinical stage at onset, *n* (%)				0.276	1.000
Ocular*	22 (81.5)	4 (50.0)	10 (58.8)		
Generalized**	5 (18.5)	4 (50.0)	7 (41.2)		
Bulbar dysfunction, *n* (%)	5 (18.6)	5 (62.5)	8 (47.1)	0.040	0.673
Myasthenia crisis, *n* (%)	4 (14.8)	3(37.5)	7 (41.2)	0.040	0.860
MGFA classification at maximum worsening, *n* (%)				0.034	0.322
I	14 (51.9)	2 (25.0)	4 (23.5)		
II	8 (29.6)	1 (12.5)	6 (35.3)		
III	5 (18.5)	2 (25.0)	5 (29.4)		
IV	0 (0.0)	1 (12.5)	1 (5.9)		
V	0 (0.0)	2 (25.0)	1 (5.9)		
Mean duration before the max MGFA occur, months	14.97	11.33	14.76	0.985	0.552
Autoantibody abnormality, *n* (%)	9 (33.3)	3 (37.5)	12 (70.6)	0.015	0.194

*AChR-MG* acetylcholine receptor antibody-positive myasthenia gravis, *DSP-MG* double-seropositive myasthenia gravis, *MGFA* Myasthenia Gravis Foundation of America, *MuSK-MG* muscle-specific tyrosine kinase antibody-positive myasthenia gravis

^*^It included those who were with the onset of ocular muscle and maintain as ocular MG during the follow-up period

^**^It included those who were with the onset of generalized muscle, as well as those with the onset of ocular muscle and then developed into generalize MG during the follow-up period

### Characteristics of DSP-MG vs MuSK-MG

There were no significant differences between DSP-MG and MuSK-MG in terms of demographics, clinical features, treatment, and prognosis (Table 2).

**Table 2:** Treatment and prognosis of AChR-MG, MuSK-MG, and DSP-MG patients

	AChR-MG (*n* = 27)	MuSK-MG (*n* = 8)	DSP-MG (*n* = 17)	P1 value (AChR-MG vs DSP-MG)	P2 value (MuSK-MG vs DSP-MG)
Treatment				< 0.001	0.475
PYR	2 (7.4)	2 (25.0)	0 (0)		
PYR + PRE	24 (88.9)	2 (25.0)	7 (41.2)		
PYR + IMM	1 (3.7)	4 (50.0)	10 (58.8)		
IVIG	3 (11.1)	3 (37.5)	3 (17.6)	0.538	0.344
Plasma exchange	0 (0.0)	0 (0.0)	0 (0.0)	1.000	1.000
Prognosis				0.001	0.628
CSR	2 (7.4)	0 (0.0)	0 (0.0)		
PR	15 (55.6)	2 (25.0)	2 (11.8)		
MM	10 (37.0)	6 (75.0)	15 (88.2)		

*AChR-MG* acetylcholine receptor antibody-positive myasthenia gravis, *CSR* complete stable remission, *DSP-MG* double-seropositive myasthenia gravis, *IMM* immunosuppressant, *IVIG* intravenous immunoglobulin, *MM* minimal manifestations, *MuSK-MG* muscle-specific tyrosine kinase antibody-positive myasthenia gravis, *PR* pharmacologic remission, *PRE* prednisone, *PYR* pyridostigmin

## Discussion

This study compared the clinical features and treatment response of 3 subtypes of MG at a hospital in southern China. We found that in both respects, DSP-MG was more similar to MuSK-MG than to AChR-MG, leading us to conclude that DSP-MG in southern China is a subtype of MuSK-MG.

Bulbar dysfunction and respiratory weakness are typical features of MuSK-MG [3, 4]. In our study, almost half of DSP-MG patients showed bulbar dysfunction, including dysarthria and dysphagia. Additionally, most of these patients experienced myasthenia crisis, which occurs in MuSK-MG. The comparison between DSP-MG and MuSK-MG patients revealed no significant differences, supporting our conclusion that they are related diseases.

The clinical manifestations of MuSK-MG tend to be worse than those of AChR-MG [3, 4]. The MGFA classification is widely used to evaluate the severity of MG, mainly based on the affected muscle groups. The most common MGFA classification at maximal worsening in AChR-MG patients was MGFA I, which indicated that weakness was mostly restricted to the ocular muscles. In contrast, most DSP-MG patients were classified as MGFA II, and the rate was higher than in AChR-MG patients; moreover, there were more patients with MGFA III to V in the DSP-MG group than in the AChR-MG group. These results indicate that DSP-MG is associated with a more severe disease status than AChR-MG and is thus more comparable to MuSK-MG. This was underscored by the fact that there was no significant difference between DSP-MG and MuSK-MG in terms of MGFA classification.

MG is an autoimmune disease that has been linked to thyroid dysfunction and autoantibody production. MG often co-occurs with other autoimmune diseases such as Graves’ disease and systemic lupus erythematous. AChR-MG is associated with abnormal thyroid function, and about 26.5% of patients have comorbid Graves’ disease [5]. On the other hand, MuSK-MG is more likely to co-occur with autoantibody diseases such as Hashimoto thyroiditis and rheumatoid arthritis (9.6%) [5]. In our study, autoantibody production was more frequently observed in DSP-MG than in AChR-MG, but there was no significant difference between DSP-MG and MuSK-MG, suggesting that they share similar pathogenic mechanisms.

Thymoma occurs in about 10–15% patients in AChR-MG but is rare in MuSK-MG [1]; therefore, thymectomy is not recommended in the latter case as no therapeutic benefit has been demonstrated [3, 4]. The high rate of thymoma among the 17 DSP-MG patients at our hospital was inconsistent with the reported rarity of thymoma in MuSK-MG. A possible reason for this incongruity is that most of our DSP-MG patients (11/17) were also positive for ryanodine receptor and titin antibodies, which occurs at a high frequency in thymoma-associated MG [1]. Thymectomy results in satisfactory improvement in thymoma-associated DSP-MG patients; therefore, although the presentation of DSP-MG is similar to that of MuSK-MG, thymectomy should be considered when thymoma is present.

In terms of treatment response, AChR-MG patients are more sensitive to cholinesterase inhibitors whereas MuSK-MG patients often require additional therapeutic interventions such as prednisone and other immunosuppressants; this difference between the 2 subtypes of MG may be attributable to differences in pathogenic mechanisms and disease severity [1, 3, 4]. MuSK-MG also has worse prognosis than AChR-MG as a result of drug insensitivity and more aggressive disease course [1, 3, 4]. In the present study, we evaluated prognosis using the PIS, which includes complete stable remission, pharmacologic remission, and mild medicine in order of worsening prognosis. None of the DSP-MG patients in our study responded to cholinesterase inhibitors and more required immunosuppressants compared to AChR-MG patients. Moreover, the rate of pharmacologic remission was lower in DSP-MG than in AChR-MG, reflecting the poorer prognosis of the former group. However, there were no differences in treatment response or prognosis between DSP-MG and MuSK-MG.

Other patient characteristics were similar between DSP-MG and MuSK-MG including the predominance of females and the higher rates of response to repetitive nerve electrical stimulation (RNS) [6] and plasma exchange [3]. Given the small size of our study population, the higher frequency of DSP-MG in women may not be significant, while the lower positive response rates may be due to technical issues and the limited number of samples. As there were no patients in our study who were treated by plasma exchange, it is unclear whether this approach can yield a better outcome.

A systematic review of the literature [7–25] published between 2005 and 2019 revealed 28 cases of DSP-MG (Table 3). Over 70% (20/28) of DSP-MG patients were female, suggesting a trend of female dominance. After removing 10 items with missing data, the initial site of DSP-MG development was in most cases the ocular (16/18) and bulbar (7/18) muscles; after removing 5 items, MGFA IIb was the most common clinical classification (8/23), indicating that bulbar weakness is a feature of DSP-MG patients. All of these features are similar to those of MuSK-MG. For thymus imaging, 4 of the remaining 9 cases had a normal thymus whereas hyperplasia and thymoma were observed in 3 and 2 cases, respectively. Previous studies have shown that MuSK-MG presents with either a normal or atrophied thymus [3–5], while thymus hyperplasia and thymoma are more common in AChR-MG [5]. Therefore, it is difficult to identify the DSP subgroup of MG based on thymus imaging. Electromyography results were mostly abnormal (9/14), which may be related to the high rate of positive response observed in MuSK-MG [6]. In terms of treatment and prognosis [2], pyridostigmin was effective in 2/20 cases, while the remaining 18 cases were treated with a combination of prednisone or immunosuppressants, intravenous immunoglobulin, or plasma exchange. Nine patients showed clinical improvement; 8 showed a worsening of symptoms; 3 improved after plasma exchange; and 1 responded to rituximab. Taken together, these observations underscore the similarity of DSP-MG to MuSK-MG [3, 4].

**Table 3:** Supplemental data of clinical characteristics

	AChR-MG (*n* = 27)	MuSK-MG (*n* = 8)	DSP-MG (*n* = 17)	P1 value (AChR-MG vs DSP-MG)	P2 value (MuSK-MG vs DSP-MG)
Thymus type, *n* (%)				0.355	0.230
Thymoma	7 (26.0)	0 (0.0)	5 (29.4)		
Hyperplasia	10 (37.0)	7 (87.5)	9 (53.0)		
Thymic atrophy	10 (37.0)	1 (12.5)	3 (17.6)		
RNS positive, *n* (%)	8 (29.6)	3 (37.5)	10 (58.8)	0.060	1.000
Abnormal thyroid function, *n* (%)	5 (18.5)	1 (12.5)	3 (17.6)	1.000	1.000

*AChR-MG* acetylcholine receptor antibody-positive myasthenia gravis, *DSP-MG* double-seropositive myasthenia gravis, *MuSK-MG* muscle-specific tyrosine kinase antibody-positive myasthenia gravis, *RNS* repetitive nerve electrical stimulation

In conclusion, although our study was limited by a small sample size, the results of our analyses and the literature search suggest that DSP-MG is a subtype of MuSK-MG. These findings can facilitate the diagnosis and treatment of DSP-MG in order to achieve better clinical outcomes (Table 4).

**Table 4:** Characteristics of DSP-MG in the literature

Number	Reference	Country	Age of onset (years)	Sex	Region of onset	MGFA	EMG	Treatment	Prognosis
1	7	Spain	45	F	Ocular, bulbar	IIb	Nor	PRE, IVIG	Worse, improved with rituximab
2	8	China	42	M	Ocular	I	Abn	PYR	Improved
3	9	Greece	71	F	Ocular	I	Abn	PYR, PRE	Improved
4	9	Greece	52	M	NA	NA	NA	NA	NA
5	9	Greece	43	M	NA	NA	NA	NA	NA
6	9	Greece	NA	F	NA	NA	NA	NA	NA
7	9	Greece	NA	F	NA	NA	NA	NA	NA
8	10	Greece	19	F	Ocular	IIIb	NA	PYR, PRE, PLEX	Worse, improved with PLEX
9	12	Sweden	61	M	Ocular	I	Abn	NA	NA
10	12	Sweden	24	F	NA	IVa	NA	NA	NA
11	12	Sweden	45	F	NA	IIa	Abn	PYR, PRE	NA
12	12	Sweden	36	F	NA	IIIa	NA	PRE, PLEX	NA
13	12	Sweden	42	F	NA	IIa	NA	PYR, PRE	NA
14	13	Germany	62	M	Ocular, bulbar	IIb	Abn	PYR, PRE, IVIG, PLEX	Worse, improved with PLEX
15	14	Poland	13	F	Ocular, limb	IIIa	Abn	PRE, IMM	Worse
16	15	UK	13	F	Ocular, bulbar	IVb	NA	PRE, PLEX	Worse
17	16	Greece	35	F	Ocular, bulbar	IIb	Abn	PYR, PRE	Improved
18	17	India	23	F	Ocular, bulbar	IIb	Abn	PYR	Improved
19	18	USA	2	F	Ocular	IIIa	Abn	PYR, PRE, IVIG	Worse
20	19	China	75	M	Bulbar, limb	IIb	Nor	PYR, PRE, IVIG	Worse
21	20	China	51	F	Ocular	I	NA	PYR, PRE, IVIG	Improved
22	21	USA	41	F	Ocular	IIb	NA	PYR, PRE, IVIG	Improved
23	22	USA	2	F	Bulbar, limb	NA	NA	PYR, PRE, IMM	Improved
24	23	USA	10	F	Ocular	IVa	Nor	PYR, PLEX	Worse
25	24	China	NA	M	NA	IIa	NA	NA	Worse, improved with PLEX
26	24	China	NA	F	NA	IVb	NA	NA	NA
27	25	China	59	M	Ocular	IIb	Nor	PYR, PRE	Improved
28	25	China	49	F	Ocular	IIb	Nor	PYR, PRE	Improved

*Abn* abnormal, *EMG* electromyography, *F* female, *IMM* immunosuppressant, *IVIG* intravenous immunoglobulin, *M* male, *NA* not available, *Nor* normal, *PLEX* plasma exchange, *PRE* prednisone, *PYR* pyridostigmin

## Abbreviations

AChR: acetylcholine receptor
AChRAb: acetylcholine receptor antibody
AChR-MG: acetylcholine receptor antibody-positive myasthenia gravis
CSR: complete stable remission
DSP-MG: double-seropositive myasthenia gravis
IMM: immunosuppressant
IVIG: intravenous immunoglobulin
MG: myasthenia gravis
MGFA: Myasthenia Gravis Foundation of America
MuSK: muscle-specific tyrosine kinase
MuSKAb: muscle-specific tyrosine kinase antibody
MuSK-MG: muscle-specific tyrosine kinase antibody-positive myasthenia gravis
MM: minimal manifestations
PIS: prognostic immune score
PR: pharmacologic remission
PRE: prednisone
PYR: pyridostigmin
PLEX: plasma exchange
RNS: repetitive nerve electrical stimulation
